# The laminin-derived peptide YIGSR (Tyr–Ile–Gly–Ser–Arg) inhibits human pre-B leukaemic cell growth and dissemination to organs in SCID mice

**DOI:** 10.1038/sj.bjc.6690618

**Published:** 1999-08

**Authors:** N Yoshida, E Ishii, M Nomizu, Y Yamada, S Mohri, N Kinukawa, A Matsuzaki, K Oshima, T Hara, S Miyazaki

**Affiliations:** Department of Pediatrics, Saga Medical School, Saga, Japan; Division of Pediatrics, Hamanomachi Hospital, 3-5-27 Maizuru, Chuo-ku, Fukuoka 810-8539, Japan; Laboratory Animal Center and Departments of Medical Informatics and Pediatrics, Faculty of Medicine, Kyushu University, Fukuoka, Japan; Department of Pathology, Fukuoka University, Fukuoka, Japan; Craniofacial Developmental Biology and Regeneration Branch, National Institute of Dental and Craniofacial Research, National Institutes of Health, Bethesda, MD 20892, USA

**Keywords:** laminin, multimeric YIGSR peptide, SCID mice, tumour metastasis, leukaemia cells

## Abstract

The YIGSR (Tyr–Ile–Gly–Ser–Arg) laminin β1 chain sequence has an inhibitory effect on tumour growth and the metastasis of melanoma and fibrosarcoma cells. In the present study, we investigated whether the multimeric YIGSR peptide (Ac-Y16) has an anti-proliferative effect and/or prevents the metastasis of human pre-B acute lymphoblastic leukaemia cells (NALM6) in severe combined immune deficient (SCID) mice. In in vitro studies, Ac-Y16 significantly inhibited leukaemic cell colony formation and the invasion of NALM6 cells in a Matrigel-based assay. The tumour growth and leukaemic infiltration in peripheral tissues were also analysed in SCID mice 9 weeks after NALM6, Matrigel and Ac-Y16 were subcutaneously co-injected. The weight of the subcutaneous tumours was significantly suppressed by Ac-Y16 in a dose-dependent manner. Flow cytometry analysis showed that the leukaemic infiltration was significantly inhibited in all organs with 1.5–2.0 mg of Ac-Y16. Leukaemic infiltrations in the brain were inhibited with 0.5 mg of Ac-Y16, and those in brain and bone marrow were also inhibited with 1.0 mg of Ac-Y16. With Ac-S16, a control-scrambled peptide, the only significant inhibition of the leukaemic infiltration was observed in bone marrow at a much higher dose. These data suggest that the multimeric YIGSR peptide can inhibit the tumour growth and metastasis of leukaemic cells and may be useful as a potential therapeutic reagent for leukaemic infiltrations. © 1999 Cancer Research Campaign


*British Journal of Cancer* (1999) **80**(12), 1898–1904
© 1999 Cancer Research Campaign
Article no. bjoc.1999.0618

### The laminin-derived peptide YIGSR (Tyr–Ile–Gly–Ser–Arg) inhibits human pre-B leukaemic cell growth and dissemination to organs in SCID mice

**N Yoshida[1], E Ishii[2], M Nomizu[7], Y Yamada[7], S Mohri[3], N Kinukawa[4], A Matsuzaki[5], K Oshima[6], T Hara[5] and S Miyazaki[1]**

[1]Department of Pediatrics, Saga Medical School, Saga, Japan; [2]Division of Pediatrics, Hamanomachi Hospital, 3-5-27 Maizuru, Chuo-ku, Fukuoka 810-8539, Japan; [3]Laboratory Animal Center and Departments of [4]Medical Informatics and [5]Pediatrics, Faculty of Medicine, Kyushu University, Fukuoka, Japan; [6]Department of Pathology, Fukuoka University, Fukuoka, Japan; [7]Craniofacial Developmental Biology and Regeneration Branch, National Institute of Dental and Craniofacial Research, National Institutes of Health, Bethesda, MD 20892, USA

**Summary** The YIGSR (Tyr–Ile–Gly–Ser–Arg) laminin β1 chain sequence has an inhibitory effect on tumour growth and the metastasis of melanoma and fibrosarcoma cells. In the present study, we investigated whether the multimeric YIGSR peptide (Ac-Y16) has an anti-proliferative effect and/or prevents the metastasis of human pre-B acute lymphoblastic leukaemia cells (NALM6) in severe combined immune deficient (SCID) mice. In in vitro studies, Ac-Y16 significantly inhibited leukaemic cell colony formation and the invasion of NALM6 cells in a Matrigel-based assay. The tumour growth and leukaemic infiltration in peripheral tissues were also analysed in SCID mice 9 weeks after NALM6, Matrigel and Ac-Y16 were subcutaneously co-injected. The weight of the subcutaneous tumours was significantly suppressed by Ac-Y16 in a dose-dependent manner. Flow cytometry analysis showed that the leukaemic infiltration was significantly inhibited in all organs with 1.5–2.0 mg of Ac-Y16. Leukaemic infiltrations in the brain were inhibited with 0.5 mg of Ac-Y16, and those in brain and bone marrow were also inhibited with 1.0 mg of Ac-Y16. With Ac-S16, a control-scrambled peptide, the only significant inhibition of the leukaemic infiltration was observed in bone marrow at a much higher dose. These data suggest that the multimeric YIGSR peptide can inhibit the tumour growth and metastasis of leukaemic cells and may be useful as a potential therapeutic reagent for leukaemic infiltrations.

**Keywords:** laminin; multimeric YIGSR peptide; SCID mice; tumour metastasis; leukaemia cells

Tumour cell invasion and metastatic spread to peripheral organs have been found to proceed in three distinct stages: (1) the attachment of tumour cells to the basement membrane via cell surface receptors such as integrins and proteoglycans; (2) the degradation of basement membrane components, which is mediated by locally secreted enzymes; and (3) the migration of tumour cells into the digested matrix (Kramer et al, 1986; Albini et al, 1987; Aznavoorian et al, 1993). Mature and neoplastic lymphoid cells circulate between blood and lymphoid organs by extravasation through both the endothelium and the basement membrane around capillaries, and then by migration along the extracellular matrix in the perivascular space (Segat et al, 1994). Matrigel, which is composed of basement membrane components including laminin, collagen type IV, proteoglycans and several growth factors, has been shown to promote the growth of leukaemic cells in xenograft models (Cavallo et al, 1991; Sterling-Levis et al, 1993). We have previously demonstrated the ability of Matrigel to promote tumour formation and dissemination to peripheral organs in severe combined immune deficient (SCID) mice using pre-B acute lymphoblastic leukaemia (ALL) cells (Ishii et al, 1995). Matrigel may provide a stromal-like support to leukaemic cells. The interaction between leukaemic cells and the matrix proteins via integrins or other cell surface receptors seems to be critical for the growth and dissemination of leukaemia in vivo.

Laminin-1, the major component of basement membrane and Matrigel, promotes cell adhesion, collagenase IV production, cell motility, and tumour growth and metastasis (Martin and Timpl, 1987; Timpl, 1989; Beck et al, 1990). Several active sites on laminin-1 have been identified using proteolytic fragments and synthetic peptides (Yamada and Kleinman, 1992). The peptide YIGSR (Tyr–Ile–Gly–Ser–Arg) comprised of residues 929–933 on the β1 chain has been found to inhibit tumour growth and metastasis (Graf et al, 1987; Iwamoto et al, 1987; Fridman et al, 1990). Both polymerized and polyethylene glycol-conjugated YIGSR peptides significantly enhanced the inhibitory effect of tumour metastasis (Murata et al, 1989; Kawasaki et al, 1991). In addition, when prepared as a multimeric peptide (Tam, 1988), YIGSR was shown to be a potent inhibitor of melanoma cell growth and metastasis (Nomizu et al, 1993). Multimeric YIGSR peptide was also shown to promote the apoptosis of fibrosarcoma cells, but not that of colon adenocarcinoma cells (Kim et al, 1994), suggesting cell-type specificity.

In the present study, we examined whether the multimeric YIGSR peptide has an anti-proliferative effect and/or prevents the metastasis of human pre-B ALL cells (NALM6) in SCID mice. We found that the multimeric YIGSR peptide significantly inhibited tumour growth and leukaemic infiltration in various organs.

*Received 30 June 1998*
*Revised 3 March 1999*
*Accepted 4 March 1999*

*Correspondence to:* E Ishii

### MATERIALS AND METHODS

### Synthesis of peptides

The multimeric YIGSR peptide (Ac-Y16), ($CH_3CO$–Tyr–Ile–Gly–Ser–Arg–Gly)$_{16}$–Lys$_8$–Lys$_4$–Lys$_2$–Lys–Gly ((Ac-YIGSRG)$_{16}$-$K_8K_4K_2$KG), was synthesized as described previously (Nomizu et al, 1993). The peptide has 16 copies of the YIGSR sequence on a branched lysine tree and has a molecular weight of approximately 10 000. A scrambled multimeric peptide (Ac-S16), (Ac-GYSRIG)$_{16}K_8K_4K_2$KG, was also synthesized as a control. The molecular weight of Ac-S16 is the same as that of AC-Y16. These peptides were purified by reverse-phase high-performance liquid chromatography. In this study, different amounts of Ac-Y16 (from 0.5 mg to 2.0 mg) were tested to examine the dose efficacy of the peptide. As a control peptide, 1.5 mg of Ac-S16 was also used with leukaemic cells and Matrigel.

### Colony assay

The effect of Ac-Y16 on the colony formation of leukaemic cells was examined. NALM6 cells ($1 \times 10^6$) were mixed with Matrigel and 0.5–2.0 mg of Ac-Y16 or 1.5 mg of Ac-S16, and $1 \times 10^4$ cells were incubated in the presence of methylcellulose as previously reported (Nakahata and Ogawa, 1982; Imai et al, 1991). Briefly, 1 ml of culture mixture containing leukaemic cells, Matrigel, Ac-Y16 or Ac-S16, α-MEM (ICN Biomedicals, Costa Mesa, CA, USA), 0.9% methylcellulose (Shin-etsu Chemical Co, Tokyo, Japan), 25% fetal bovine serum (FBS), 1% deionized bovine serum albumin (Sigma Chemical Co., St Louis, MO, USA), and $5 \times 10^{-5}$ mol $l^{-1}$ 2-mercaptoethanol (Sigma), was plated in a 35 mm Lux standard non-tissue culture dish (Nunc, Inc., Naperville, IL, USA) and incubated for 7 days at 37°C in a humidified atmosphere flushed with 5% carbon dioxide, 5% oxygen and 90% $N_2$. The number of leukaemic colonies (defined as > 40 cells) was counted using an inverted microscope. All experiments were performed using quadruplicate samples.

### Invasion assay

The invasion of leukaemic cells and its inhibition by Ac-Y16 or Ac-S16 were analysed in the Matrigel-based assay according to the previous methods (Janiak et al, 1994; Matsuzaki et al, 1996). The Matrigel invasion chamber containing an 8-micron pore size membrane coated with Matrigel was used for the study (Becton Dickinson, Bedford, MA, USA). Lower compartments of the chambers were filled with 500 µl of RPMI-1640 medium. The leukaemic cells ($1 \times 10^6$) were mixed with 0.5–1.5 mg of Ac-Y16 or 1.5 mg of Ac-S16 in the final volume of 1 ml. Then 200 µl of the cells were placed in the upper compartment ($0.2 \times 10^6$ cells per chamber). After incubation for 4 h at 37°C, the number of cells that had migrated into the lower compartment of each chamber was counted to determine the percentage of invasion. All experiments were performed using three chambers for each sample.

### Engraftment of pre-B leukaemic cells in SCID mice

NALM6, a human pre-B ALL cell line (Uckun et al, 1992), was maintained in RPMI-1640 medium with 10% FBS (Gibco, Grand Island, NY, USA). The SCID mice were bred and maintained in defined flora colonies (Faculty of Medicine, Kyushu University, Fukuoka, Japan). The engraftment of NALM6 cells in SCID mice was performed as described previously (Ishii et al, 1995). Briefly, NALM6 cells ($2 \times 10^6$) and 2 mg of Matrigel (Becton Dickinson) were co-injected subcutaneously (s.c.) with 0.5–2.0 mg of Ac-Y16 or 1.5 mg of Ac-S16 in 8–10-week-old SCID mice. After 9 weeks, the mice were sacrificed and the tumour weights in mice with or without either Ac-Y16 or As-S16 were measured. The s.c. tumour, spleen, liver, lung, kidney, brain and bone marrow (BM) in each mouse were excised and minced with a 40-mesh screen. The cell suspensions of each organ were washed several times with medium and then subjected to flow cytometry. The number of mice analysed in the study was 5–8 in each group. Total RNA was extracted from these tissues for analysis by the reverse transcriptase polymerase chain reaction (RT-PCR) method.

### Flow cytometry

CD10- and CD19-positive human cells in each organ were analysed with monoclonal antibodies by two-colour direct immunofluorescence to measure infiltration by leukaemic cells. Cell suspensions from tumours, spleen, liver, lung, kidney and brain were prepared by a tissue grinder. A cell suspension from BM was also prepared by gentle pipetting. Aggregated cells or residual tissues were removed by brief sedimentation. For erythrocyte lysis, each sample was diluted with $NH_4Cl$ lysis buffer and after gentle mixing kept at room temperature for 3–5 min. Aliquots of a cell suspension ($1 \times 10^6$ cells) from the primary tumours, spleen, liver, lung, kidney, brain and BM were incubated with 10 µl of fluorscein isothiocyanate (FITC)-conjugated OKB CALLA specific for CD10 (Ortho Diagnostic Systems, Raritan, NJ, USA) and 20 µl of PE-conjugated HD37 specific for CD19 (Dako, Glostrup, Denmark) to identify human leukaemic cells. Samples were then mixed gently, incubated at 4°C for 30 min and washed twice in phosphate-buffered saline (PBS). For the flow cytometric analysis, $2 \times 10^4$ cells were analysed on a FACScan (Becton Dickinson, Mountain View, CA, USA). Human and murine BM or peripheral blood cells were used for the gating of mononuclear cells. In each experiment, cells from non-transplanted mice were stained with the same antibodies, as a negative control. An isotype control antibody was also used. The percent infiltration of leukaemic cells was defined as the ratio of the number of $CD10^+CD19^+$-positive cells to that of all mononuclear cells in each organ.

### RT-PCR

Total RNA was extracted from tumour and organ homogenates (Ishii et al, 1995). cDNA was prepared by RT at 37°C for 60 min in a 50 µl mixture containing 5 µg RNA, 1 µg oligo-dT and 100 units of MMLV reverse transcriptase (Life Technology, Rockville, MD, USA). The integrity of RNA and cDNA was confirmed by the generation of a β-actin PCR product (228 bp) with primers which hybridize with both human and murine β-actin (actin-MH); 5′-CTACAATGAGCTGCGTGTGG-3′ and 5′-TAGATGGGCAC-AGTGTGGGT-3′ (Nakajima-Iijima et al, 1985). Primers specific for human β-actin (actin-H) were also prepared to detect human leukaemic cells in mouse organs. These primers were 5′-GGCCACGGCTGCTTCCAG-3′ and 5′-CATTGTGCTGGGT-GCCAGG-3′ (Nakajima-Iijima et al, 1985). The expected size of the PCR product was 295 bp. The PCR was performed with cDNA in a total volume of 50 µl, containing 1 × PCR buffer, 200 µM

*© 1999 Cancer Research Campaign* 

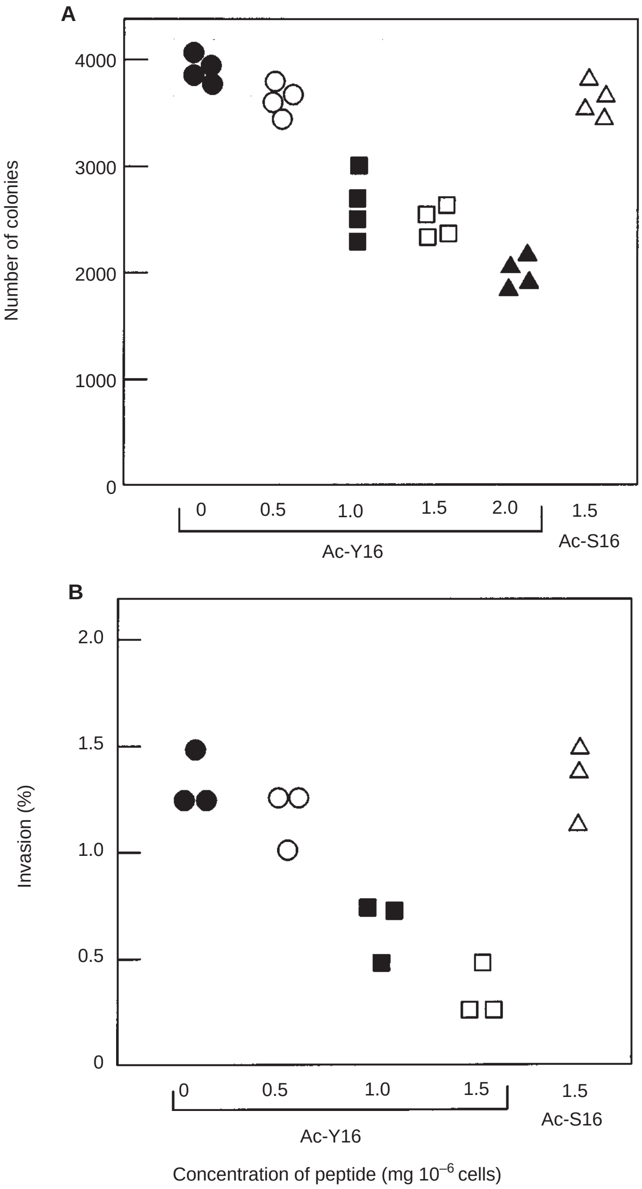

**Figure 1** Inhibitory effect of Ac-Y16 or Ac-S16 on the colony formation (**A**) and the invasive potential (**B**) of NALM6 cells. Significant inhibition by Ac-Y16 was observed in colony formation ($P < 0.01$) and invasiveness ($P < 0.05$)

dNTPs, 0.5 μg each of the 5′ and 3′ primers and 2.5 units of *Taq* polymerase (Perkin-Elmer, Norwalk, CT, USA). The amplification profile involved 30 cycles of denaturation at 94°C for 30 s, primer annealing at 53°C for 30 s and extension at 72°C for 1 min. The PCR products were resolved by electrophoresis in 2% agarose gels, stained with ethidium bromide and photographed. The reproducibility of the PCR analysis was confirmed by several independent experiments.

### Apoptosis assays

NALM6 cells ($4 \times 10^5$ ml$^{-1}$) were co-incubated with 2–50 μg of Ac-Y16 or Ac-S16 for 12 h at 37°C. After staining leukaemic cells with 10 μl of FITC-conjugated annexin V and 5 μl of propidium iodide (MEBCYTO-Apoptosis Kit, MBL, Japan) for 15 min, the number of apoptotic cells was measured by flow cytometry (van Engeland et al, 1998). We also measured the mitotic index of leukaemic cells and the number of apoptotic cells in the s.c. tumours by histological examination and/or TUNEL assays (ApoptaqPlus, Oncor, Gaithersburg, MD, USA).

### Statistical analysis

Differences in the colony formation and invasion of leukaemic cells in vitro between the test concentration groups were compared by the Kruskal–Wallis test. The tumour weight and the infiltration of leukaemic cells in mice with or without Ac-Y16 or Ac-S16 were also compared by the Kruskal–Wallis test. A multiple comparison test was performed using the method of Bonferroni adjustment when the the Kruskal–Wallis test showed $P < 0.05$ (Alt, 1982).

### RESULTS

### Effect of Ac-Y16 on the colony formation and invasion of leukaemic cells

We examined the effect of Ac-Y16 on the colony formation of leukaemic cells in vitro. NALM6 cells were incubated with Matrigel and 0.5–2.0 mg of Ac-Y16 or 1.5 mg of Ac-S16, and the number of leukaemic colonies was measured after 7 days of incubation. As shown in Figure 1A, the number of colonies was decreased with increasing amounts of Ac-Y16. A significant difference was observed between test concentration groups ($P < 0.01$). Ac-S16, the control-scrambled peptide, did not inhibit the colony formation of NALM6 cells. Thus, the Ac-Y16 peptide inhibited leukaemic cell growth in vitro.

The in vitro effect of Ac-Y16 on the invasion of leukaemic cells was also examined (Figure 1B). Approximately 1.25–1.50% of the leukaemic cells migrated through a Matrigel-coated membrane. With increasing amounts of Ac-Y16, the percentage of leukaemic cells that migrated into lower compartment was significantly decreased ($P < 0.05$). Ac-S16 showed little effect on the cell migration. These data suggest that laminin plays an important role in leukaemic cell invasion in vitro.

To examine whether Ac-Y16 induces apoptosis of leukaemic cells, NALM6 cells were co-cultured with 2–50 μg of Ac-Y16 or Ac-S16 and the number of apoptotic cells was measured by flow cytometry. The percentage of apoptotic cells was less than 3% in control leukemic cells, whereas in cells treated with Ac-Y16 10.8% at 2 μg ml$^{-1}$, 10.3% at 10 μg ml$^{-1}$ and 33.1% at 50 μg ml$^{-1}$ of Ac-Y16 were apoptotic after 12 h of treatment. Control Ac-S16 showed less apoptosis than Ac-Y16, i.e. 8.8% at 2 μg ml$^{-1}$, 8.1% at 10 μg ml$^{-1}$ and 13.5% at 50 μg ml$^{-1}$.

### Effects of the multimeric YIGSR (Ac-Y16) peptide on tumour growth and the dissemination of leukaemic cells in SCID mice

Human pre-B leukaemia NALM6 cells ($2 \times 10^6$) and Matrigel (2 mg) were injected s.c. in SCID mice with or without the multimeric YIGSR peptide (Ac-Y16). As a control, Ac-S16 was also injected with Matrigel and NALM6 cells. After 9 weeks, the growth of the primary tumour and the infiltration of leukaemic cells into the spleen, liver, lung, kidney, brain and BM were

 © 1999 Cancer Research Campaign

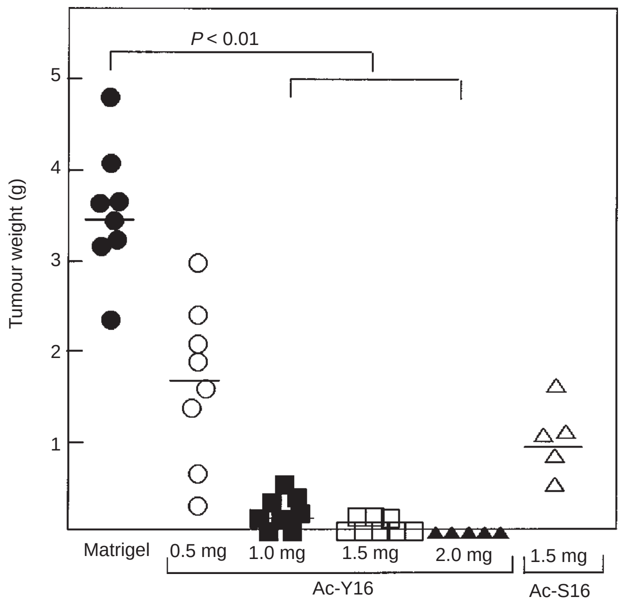

**Figure 2** Inhibition of tumour formation by Ac-Y16 and Ac-S16. Nine weeks after the injection of NALM6 cells with Matrigel and 0.5–2.0 mg of Ac-Y16 or 1.5 mg of Ac-S16, the tumour growth was analysed. The tumours decreased with the dose of Ac-Y16; a significant difference in the tumour weight was observed between mice with Matrigel alone and those with 1.0–2.0 mg of Ac-Y16. Each horizontal bar indicates the mean value of the samples analysed. The weight of the s.c. tumours differed significantly in the mice with or without Ac-Y16 or Ac-S16 ($P$ = 0.0004) (Figure 2). No tumour formation was observed in mice when 1.5 or 2.0 mg of Ac-Y16 was coinjected. One milligram of Ac-Y16 significantly inhibited tumour growth ($P < 0.01$) by more than 90%. In contrast, when 1.5 mg of Ac-S16 were co-injected with leukaemic cells, a lesser inhibitory effect on tumour growth was observed. However, these differences were not significant (Figure 2). To examine whether the inhibition by Ac-Y16 of tumour growth is due to the inhibition of cell proliferation, to cell death by apoptosis, or both, we measured the mitotic index of leukaemic cells and the number of apoptotic cells of the s.c. tumours. The mitotic index and the number of apoptotic cells were not significantly different in the s.c. tumours with and without Ac-Y16 or Ac-S16 (data not shown). These findings were confirmed by the staining with MIB-1 monoclonal antibodies to detect mitotic cells.

The infiltration of leukaemic cells in peripheral organs was assessed in a flow cytometry analysis. The flow cytometric profiles of representative samples obtained from untreated control mice and those with leukaemic cells and Matrigel are given in Figure 3. All of the organs from untreated mice showed no or very low reactivity with both the CD10 and CD19 antibodies, whereas high numbers of $CD10^+CD19^+$ cells were detected in organs from mice with leukaemic cells and Matrigel. Figure 4 shows the per cent infiltration of leukaemic cells in all organs from six different

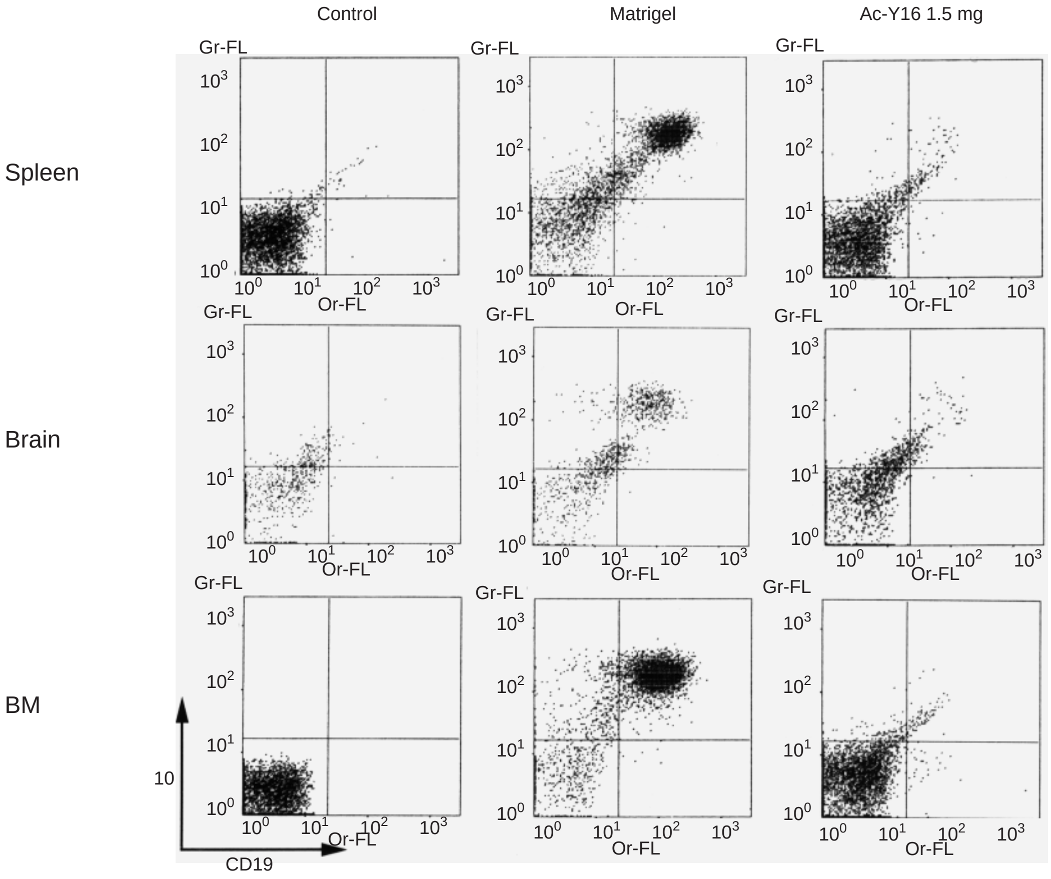

**Figure 3** The reactivity with CD10- and CD19-specific antibodies in organs from control untreated mice, mice treated with leukaemic cells and Matrigel, or those treated with leukaemic cells and Matrigel/Ac-Y16. No or very low numbers of $CD10^+CD19^+$ cells were observed in the control mice, whereas high numbers of $CD10^+CD19^+$ cells were detected in the mice with Matrigel. The infiltration of $CD10^+CD19^+$ cells was suppressed by 1.5 mg of Ac-Y16

© 1999 Cancer Research Campaign

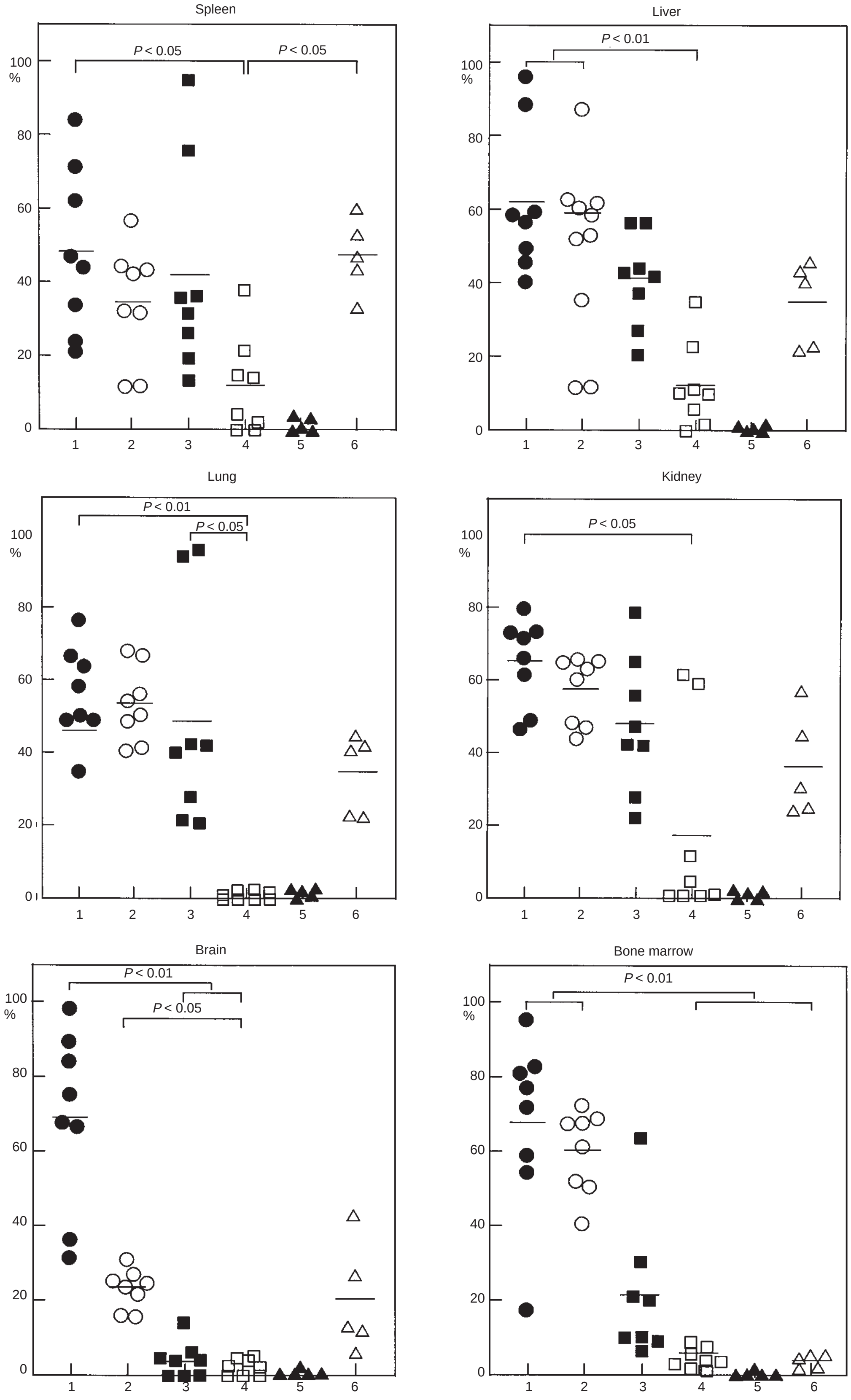

**Figure 4** Infiltration of leukaemic cells in peripheral organs. The leukaemic infiltration, defined as the per cent of $CD10^{+}CD19^{+}$ human cells in each organ, was measured by flow cytometry and plotted. Each horizontal bar shows the mean of the per cent infiltration of leukaemic cells in mice. Lane 1, mice with Matrigel ($n = 8$); lane 2, mice with Matrigel and 0.5 mg Ac-Y16 ($n = 8$); lane 3, mice with Matrigel and 1.0 mg Ac-Y16 ($n = 8$); lane 4, mice with Matrigel and 1.5 mg Ac-Y16 ($n = 8$); lane 5, mice with Matrigel and 2.0 mg AC-Y16 ($n = 5$); lane 6, mice with Matrigel and 1.5 mg Ac-S16 ($n = 5$)

*© 1999 Cancer Research Campaign*

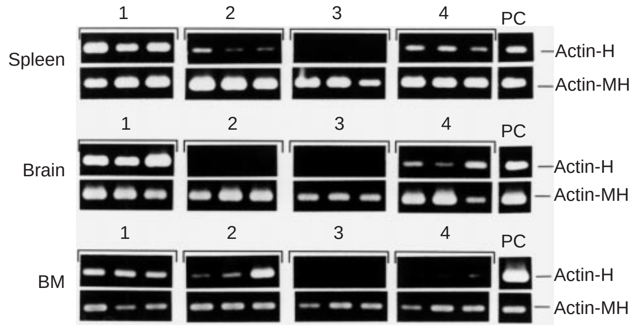

**Figure 5** Infiltration of leukaemic cells in the mouse spleen, brain and BM assessed by RT-PCR. Human β-actin mRNA (actin-H) was detected in the spleen, brain and BM of mice with Matrigel alone (lane 1), and with 1.5 mg of Ac-S16 (lane 4). In mice treated with Ac-Y16, only the spleen and BM expressed human β-actin at 1.0 mg (lane 2), whereas there was no expression of human β-actin in these organs at 1.5 mg (lane 3). Actin-MH was used as an internal control. PC, NALM6 cells as a positive control groups of mice. High levels of leukaemic infiltration were observed in the spleen, liver, lung, kidney, brain and BM of all mice with Matrigel alone. The infiltration of leukaemic cells in the mice with and without Ac-Y16 or Ac-S16 was significantly different in all organs: $P = 0.0075$ in spleen, $P = 0.0001$ in liver, $P = 0.0001$ in lung, $P = 0.0018$ in kidney, $P < 0.0001$ in brain and $P < 0.0001$ in BM. Two mg of Ac-Y16 completely suppressed the infiltration of leukaemic cells in all organs. At 1.5 mg of Ac-Y16, the leukaemic infiltration was significantly inhibited in the spleen ($P < 0.05$) and other organs ($P < 0.01$), which is also shown in Figure 3. Of all the organs examined, the brain was the most sensitive to the presence of the test peptide. Only a low infiltration of leukaemic cells was observed in the brain of mice treated with Ac-Y16 at 0.5 mg or at 1.0 mg. In contrast, with 1.5 mg of Ac-S16, a significant inhibition of leukaemic infiltration was observed only in BM ($P < 0.01$).

The dissemination of leukaemia cells was also assessed by the RT-PCR analysis of human β-actin mRNA expression in the spleen, brain and BM (Figure 5). Human β-actin mRNA was detected in the spleen, brain and BM of mice treated with Matrigel alone. In the mice treated with 1.0 mg of Ac-Y16, the expression of human β-actin mRNA was observed in only the spleen and BM; the brain showed undetectable levels of human β-actin mRNA. Mice treated with 1.5 mg of Ac-Y16 showed undetectable mRNA of β-actin in all organs. In contrast, the human β-actin mRNA was detected in mice with 1.5 mg of Ac-S16. The BM in mice treated with Ac-S16, which showed only a small infiltration of leukaemic cells by flow cytometry, also expressed human β-actin by RT-PCR. Thus, Ac-Y16 inhibited the tumour growth and dissemination of leukaemic cells into peripheral organs in a dose-dependent manner.

### DISCUSSION

The Tyr–Ile–Gly–Ser–Arg (YIGSR) sequence derived from the laminin β1 chain has been shown to inhibit tumour growth and metastasis (Graf et al, 1987; Iwamoto et al, 1987; Saiki et al, 1989; Fridman et al, 1990). It was reported that the multimeric YIGSR polypeptide greatly enhanced the inhibition of tumour growth and metastasis (Nomizu et al, 1993). Although the mechanisms of this effect of YIGSR are still not clear, recent results have suggested that apoptosis may play a role in the anti-metastatic and anti-tumour effects associated with multimeric YIGSR peptide in HT-1080 human fibrosarcoma cells (Kim et al, 1994). However, cell type-specific apoptosis by YIGSR has not been demonstrated (Kim et al, 1994). It was also reported that YIGSR reduces angiogenesis (Sakamoto et al, 1991; Iwamoto et al, 1996).

The interaction between Matrigel and leukaemic cells can also facilitate a proliferative response (Sterling-Levis et al, 1993; Ishii et al, 1995; Yan et al, 1996). As shown in a previous report (Blase et al, 1996), NALM6 expressed high levels of VLA-α3, -α4, -α5 and -α6, and VLA-β1. VLA-α6, which is usually expressed on pre-B leukaemic cells, interacts with laminin (Hynes, 1992). In fact, NALM6 cells mainly adhere to laminin, and this binding is significantly reduced by the β1 and α6 monoclonal antibodies (Blase et al, 1996). We also found that Ac-Y16 has similar activity for NALM6 cell attachment (data not shown). However, the binding site of laminin-1 for VLA-α3 and α6 integrins is the C-terminal portion (Hall et al, 1990; Tomaselli et al, 1990; Sonnenberg et al, 1991), while YIGSR has been shown to recognize 36-kDa, 38-kDa and 67-kDa cell surface proteins (Graf et al, 1987; Clement et al, 1990) and α4β1 integrin (Maeda et al, 1994). Taken together, these findings indicate that YIGSR may inhibit tumour formation and leukaemic infiltration by competing with laminin for these laminin receptors and/or integrins on leukaemic cells, thus blocking the binding of the cells to basement membrane (Iwamoto et al, 1987).

In our study, the growth and dissemination of leukaemic cells were inhibited by the multimeric YIGSR peptide in vivo and in vitro. The precise mechanism of the drug-provoked tumour inhibition is unclear. Although the direct toxicity of YIGSR peptide for leukaemic cells cannot be completely ruled out, Ac-Y16 reduced tumour growth at 0.5–1.0 mg in vitro and selectively inhibited the dissemination of leukaemic cells in vivo. Previous data also suggested that Ac-Y16 is not cytotoxic in vivo (Iwamoto et al, 1996). In the present study, the high dose (1.5–2.0 mg) of Ac-Y16 clearly inhibited the tumour formation and leukaemic infiltration in all peripheral organs, compared with the same dose of Ac-S16, a scrambled multimeric peptide, which showed only a weak inhibitory effect on leukaemic infiltration. Apoptosis of NALM6 cells were induced by Ac-Y16 in cultures, whereas the number of apoptotic cells in the s.c. tumours was not increased by Ac-Y16. Although this discrepancy is not clear, it is possible that the sensitivity of the apoptosis assays may be different between cell cultures and in vivo. Another possibility is that Ac-Y16-mediated apoptosis may occur in much earlier stages after the inoculation of NALM6 cells with Ac-Y16 in SCID mice. Our apoptosis assays were performed at 12 h after the incubation and at this late stage apoptotic cells may not be present and could not be detected. Alternatively, Ac-Y16 may be more potent in inhibiting tumour cell proliferation or inducing necrosis of tumour cells than apoptosis in vivo.

In the previous study, the proliferation of HT-1080 cells was markedly decreased by Ac-Y16 at 60–100 $\mu g\ ml^{-1}$, while only a small effect was observed at 30 $\mu g\ ml^{-1}$ (Kim et al, 1994). Proliferation of SW480 cells was reduced at 100 $\mu g\ ml^{-1}$, but had no effect at 30 $\mu g\ ml^{-1}$ (Kim et al, 1994). In our study, the colony formation of leukaemic cells was partially suppressed by Ac-Y16 at 0.5–2.0 mg per $10^6$ cells (5–20 $\mu g\ ml^{-1}$). Although assays used for these two studies are different, their data suggest that the inhibitory activity of Ac-Y16 varies with different cell types.

Leukaemic cells usually spread from bone marrow or the tumour burden to peripheral organs as overt leukaemia. In order to

© 1999 Cancer Research Campaign disseminate, leukaemic cells enter the circulatory system by crossing the endothelium and the basement membrane. The multimeric YIGSR peptide may inhibit the spreading of leukaemic cells to the vascular endothelium, by blocking leukaemic cell binding to laminin. Only one leukaemic cell line was used in the present study; further analyses is necessary to examine the inhibitory activity of Ac-Y16 for primary leukaemic cells from patients.

### ACKNOWLEDGEMENTS

We thank Fumio Nagumo (Central Laboratory, Saga Medical School) and Seiko Yoshidomi (Laboratory Division, Saga Prefectural Hospital) for their assistance with the flow cytometry. We also thank Benjamin Weeks (Adelphi University) for his critical reading of the manuscript. This work was supported by a grant from the Idiopathic Disorder of Hematopoietic Organs Research Committee of Japan.

### REFERENCES

Albini A, Iwamoto Y, Kleinman HK, Martin GR, Aaronson SA, Kozlowski JM and McEwan RN (1987) A rapid in vitro assay for quantitating the invasion potential of tumor cells. *Cancer Res* **47**: 3239–3245

Alt FB (1982) *Bonferroni Inequalities and Intervals. Encyclopedia of Statistical Sciences* Vol. 1, pp. 294–300. John Wiley & Sons: New York

Aznavoorian S, Murphy AN, Stetler-Stevenson WG and Liotta LA (1993) Molecular aspects of tumor cell invasion and metastasis. *Cancer* **71**: 1368–1383

Beck K, Hunter I and Engel J (1990) Structure and function of laminin: anatomy of multidomain glycoprotein. *FASEB J* **4**: 148–160

Blase L, Merling A, Engelmann S, Moller P and Schwartz-Albiez R (1996) Characterization of cell surface-expressed proteochondroitin sulfate of pre-B Nalm-6 cells and its possible role in laminin adhesion. *Leukemia* **10**: 1000–1011

Cavallo F, Riccardi C, Forni M, Pericle F, Bosco MC, Giovarelli M, Soleti A and Forni G (1991) Growth and dissemination of human malignant lymphoblasts in immunosuppressed nu/nu mice. *Nat Immun Cell Growth Regul* **10**: 256–264

Clement B, Segui-Real B, Savagner P, Kleinman HK and Yamada Y (1990) Hepatocyte attachment to laminin is mediated through multiple receptors. *J Cell Biol* **110**: 185–192

Fridman R, Giaccone G, Kanemoto T, Martin GR, Gazdar AF and Mulshine JL (1990) Reconstituted basement membrane (matrigel) and laminin can enhance the tumorigenicity and the drug resistance of small lung cancer cell lines. *Proc Natl Acad Sci USA* **87**: 6698–6702

Fridman R, Kibby MC, Royce LS, Zain M, Sweeney TM, Jicha DL, Yannelli JR, Martin GR and Kleinman HK (1991) Enhanced tumor growth of both primary and established human and murine tumor cells in athymic mice after coinjection with matrigel. *J Natl Cancer Inst* **83**: 769–774

Graf J, Iwamoto Y, Sasaki M, Martin GR, Kleinman HK, Robey FA and Yamada Y (1987) Identification of an amino acid sequence in laminin mediating cell attachment, chemotoxis, and receptor binding. *Cell* **48**: 989–996

Hall DE, Reichardt LF, Crowley E, Holley B, Moezzi H, Sonnenberg A and Damsky CH (1990) The α 1/β 1 and α 6/β 1 integrin heterodimers mediate cell attachment to distinct sites on laminin. *J Cell Biol* **110**: 2175–2184

Hynes RO (1992) Integrins: versatility, modulation, and signaling in cell adhesion. *Cell* **69**: 11–25

Imai T, Koike K, Kubo T, Kikuchi T, Amano Y, Takagi M, Okamura N and Nakahata T (1991) Interleukin-6 supports human megakaryocytic proliferation and differentiation in vitro. *Blood* **78**: 1969–1974

Ishii E, Greaves A, Grunberger T, Freedman MH and Letarte M (1995) Tumor formation by a pre-B leukemia cell line in scid mice is associated with CD10 induction and is promoted by Matrigel. *Leukemia* **9**: 175–184

Iwamoto Y, Robey FA, Graf J, Sasaki M, Kleinman HK, Yamada Y and Martin GR (1987) YIGSR, a synthetic laminin pentapeptide, inhibits experimental metastasis formation. *Science* **238**: 1132–1134

Iwamoto Y, Nomizu M, Yamada Y, Ito Y, Tanaka K and Sugioka Y (1996) Inhibition of angiogenesis, tumour growth and experimental metastasis of human fibrosarcoma cells HT1080 by a multimeric form of the laminin sequence Tyr–Ile–Gly–Ser–Arg (YIGSR). *Br J Cancer* 589–595

Janiak M, Hashmi HR and Janowska-Wieczorek A (1994) Use of the Matrigel-based assay to measure the invasiveness of leukemic cells. *Exp Hematol* **22**: 559–565

Kath R, Jambrosic JA, Holland L, Rodeck U and Herlyn M (1991) Development of invasive and growth factor-independent cell variants from primary melanomas. *Cancer Res* **51**: 2205–2211

Kawasaki K, Namikawa M, Murakami T, Mizuta T, Iwai Y, Hama T and Mayumi T (1991) Amino acids and peptides. XIV. Laminin related peptides and their inhibitory effect on experimental metastasis formation. *Biochm Biophys Res Commun* **174**: 1159–1162

Kim WH, Schnaper W, Nomizu M, Yamada Y and Kleinman HK (1994) Apoptosis in human fibrosarcoma cells is induced by a multimeric synthetic Tyr-Ile-Gly-Ser-Arg (YIGSR)-containing polypeptide from laminin. *Cancer Res* **54**: 5005–5010

Kramer RH, Bensch KG and Wong J (1986) Invasion of reconstituted basement membrane matrix by metastatic human tumor cells. *Cancer Res* **46**: 1980–1989

Matsuzaki A and Janowska-Wieczorek A (1996) Unstimulated human acute myelogenous leukemia blasts secrete matrix metalloproteinases. *Cancer Res Clin Oncol* **123**: 100–106

Maeda T, Titani K and Sekiguchi K (1994) Cell-adhesive activity and receptor-binding specificity of the laminin-derived YIGSR sequence grafted onto Staphylococcal protein A. *J Biochem* (Tokyo) **115**: 182–189

Martin GR and Timpl R (1987) Laminin and other basement membrane components. *Annu Rev Cell Biol* **3**: 57–85

Murata J, Saiki I, Azuma I and Nishi N (1989) Inhibitory effect of a synthetic polypeptide, poly (Tyr–Ile–Gly–Ser–Arg), on the metastatic formation of malignant tumor cells. *Int J Biol Macromol* **11**: 97–99

Nakahata T and Ogawa M (1982) Hemopoietic colony-forming cells in umbilical cord blood with extensive capability to generate mono- and multipotential hemopoietic progenitors. *J Clin Invest* **70**: 1324–1328

Nakajima-Iijima S, Hamada H, Reddy P and Kakunaga T (1985) Molecular structure of the human cytoplasmic β-actin gene: interspecies homology of sequences in the introns. *Proc Natl Acad Sci USA* **82**: 6133–6137

Nomizu M, Yamamura K, Kleinman HK and Yamada Y (1993) Multimeric forms of Tyr–Ile–Gly–Ser–Arg (YIGSR) peptide enhance the inhibition of tumor growth and metastasis. *Cancer Res* **53**: 3459–3461

Saiki I, Murata J, Iida J, Nishi N, Sugimura K and Azuma I (1989) The inhibition of murine lung metastasis by synthetic polypeptide (poly(Arg–Gly–Asp) and poly(Tyr–Ile–Gly–Ser–Arg)) with a core sequence of cell adhesion molecules. *Br J Cancer* **59**: 194–197

Sakamoto N, Iwahana M, Tanaka NG and Osada Y (1991) Inhibition of angiogenesis and tumor growth by a synthetic laminin peptide. *Cancer Res* **51**: 903–906

Segat D, Pucillo C, Marotta G, Perris R and Colombatti A (1994) Differential attachment of human neoplastic B cells to purified extracellular matrix molecules. *Blood* **83**: 1586–1594

Sonnenberg A, Gehlsen KR, Aumailley M and Timpl R (1991) Isolation of $\alpha_6\beta_1$ integrins from platelets and adherent cells by affinity chromatography on mouse laminin fragment E8 and human laminin pepsin fragment. *Exp Cell Res* **197**: 234–244

Sterling-Levis K, White L, Trickett AE, Gramacho C, Pittman SM and Tobias V (1993) Heterotransplantation of early B-lineage acute lymphoblastic leukemia using a solubulized attachment matrix (matrigel). *Cancer Res* **53**: 1222–1225

Tam JP (1988) Synthetic peptide vaccine design: synthesis and properties of a high-density multiple antigenic peptide system. *Proc Natl Acad Sci USA* **85**: 5409–5413

Timpl R (1989) Structure and biological activity of basement membrane proteins. *Eur J Biochem* **180**: 148–502

Tomaselli KJ, Hall DE, Flier LA, Gehlsen KR, Turner DC, Carbonetto S, Reichardt LF (1990) A neuronal cell line (PC12) expresses two β1-class integrins – $\alpha_1\beta_1$ and $\alpha_3\beta_1$ – that recognize different neurite outgrowth-promoting domains in laminin. *Neuron* **5**: 651–662

Uckun FM, Manivel C, Arthur D, Chelstrom LM, Finnegan D, Tuel-Ahlgren L, Irvin JD, Myers DE and Gunther R (1992) In vivo efficacy of B43 (anti-CD19)-pokeweed antiviral protein immunotoxin against human pre-B cell acute lymphoblastic leukemia in mice with severe combined immunodeficiency. *Blood* **79**: 2201–2214

van Engeland M, Nieland LJ, Ramaekers FC, Schutte B, Reutelingsperger CP (1998) Annexin V-affinity assay: a review on an apoptosis detection system based on phosphatidylserine exposure. *Cytometry* **31**: 1–9

Yamada Y and Kleinman HK (1992) Functional domains of cell adhesion molecules. *Curr Opin Cell Biol* **4**: 819–823

Yan Y, Salomon O, McGuirk J, Denning D, Fernandez J, Jagiello C, Collins N, Steinherz P and O'Reilly RJ (1996) Growth pattern and clinical correlation of subcutaneously inoculated human primary acute leukemias in severe combined immunodeficiency mice. *Blood* **88**: 3137–3146

 © 1999 Cancer Research Campaign

## References

[PDF_00681] Albini A., Iwamoto Y., Kleinman H. K., Martin G. R., Aaronson S. A., Kozlowski J. M., McEwan R. N. (1987). A rapid in vitro assay for quantitating the invasive potential of tumor cells.. Cancer Res.

[PDF_00686] Aznavoorian S., Murphy A. N., Stetler-Stevenson W. G., Liotta L. A. (1993). Molecular aspects of tumor cell invasion and metastasis.. Cancer.

[PDF_00689] Beck K., Hunter I., Engel J. (1990). Structure and function of laminin: anatomy of a multidomain glycoprotein.. FASEB J.

[PDF_00691] Blase L., Merling A., Engelmann S., Möller P., Schwartz-Albiez R. (1996). Characterization of cell surface-expressed proteochondroitin sulfate of pre-B Nalm-6 cells and its possible role in laminin adhesion.. Leukemia.

[PDF_00695] Cavallo F., Riccardi C., Forni M., Pericle F., Bosco M. C., Giovarelli M., Soleti A., Forni G. (1991). Growth and dissemination of human malignant lymphoblasts in immunosuppressed nu/nu mice.. Nat Immun Cell Growth Regul.

[PDF_00699] Clément B., Segui-Real B., Savagner P., Kleinman H. K., Yamada Y. (1990). Hepatocyte attachment to laminin is mediated through multiple receptors.. J Cell Biol.

[PDF_00702] Fridman R., Giaccone G., Kanemoto T., Martin G. R., Gazdar A. F., Mulshine J. L. (1990). Reconstituted basement membrane (matrigel) and laminin can enhance the tumorigenicity and the drug resistance of small cell lung cancer cell lines.. Proc Natl Acad Sci U S A.

[PDF_00706] Fridman R., Kibbey M. C., Royce L. S., Zain M., Sweeney M., Jicha D. L., Yannelli J. R., Martin G. R., Kleinman H. K. (1991). Enhanced tumor growth of both primary and established human and murine tumor cells in athymic mice after coinjection with Matrigel.. J Natl Cancer Inst.

[PDF_00710] Graf J., Iwamoto Y., Sasaki M., Martin G. R., Kleinman H. K., Robey F. A., Yamada Y. (1987). Identification of an amino acid sequence in laminin mediating cell attachment, chemotaxis, and receptor binding.. Cell.

[PDF_00713] Hall D. E., Reichardt L. F., Crowley E., Holley B., Moezzi H., Sonnenberg A., Damsky C. H. (1990). The alpha 1/beta 1 and alpha 6/beta 1 integrin heterodimers mediate cell attachment to distinct sites on laminin.. J Cell Biol.

[PDF_00716] Hynes R. O. (1992). Integrins: versatility, modulation, and signaling in cell adhesion.. Cell.

[PDF_00718] Imai T., Koike K., Kubo T., Kikuchi T., Amano Y., Takagi M., Okumura N., Nakahata T. (1991). Interleukin-6 supports human megakaryocytic proliferation and differentiation in vitro.. Blood.

[PDF_00721] Ishii E., Greaves A., Grunberger T., Freedman M. H., Letarte M. (1995). Tumor formation by a human pre-B leukemia cell line in scid mice is enhanced by matrigel and is associated with induction of CD10 expression.. Leukemia.

[PDF_00727] Iwamoto Y., Nomizu M., Yamada Y., Ito Y., Tanaka K., Sugioka Y. (1996). Inhibition of angiogenesis, tumour growth and experimental metastasis of human fibrosarcoma cells HT1080 by a multimeric form of the laminin sequence Tyr-Ile-Gly-Ser-Arg (YIGSR).. Br J Cancer.

[PDF_00724] Iwamoto Y., Robey F. A., Graf J., Sasaki M., Kleinman H. K., Yamada Y., Martin G. R. (1987). YIGSR, a synthetic laminin pentapeptide, inhibits experimental metastasis formation.. Science.

[PDF_00731] Janiak M., Hashmi H. R., Janowska-Wieczorek A. (1994). Use of the Matrigel-based assay to measure the invasiveness of leukemic cells.. Exp Hematol.

[PDF_00733] Kath R., Jambrosic J. A., Holland L., Rodeck U., Herlyn M. (1991). Development of invasive and growth factor-independent cell variants from primary human melanomas.. Cancer Res.

[PDF_00736] Kawasaki K., Namikawa M., Murakami T., Mizuta T., Iwai Y., Hama T., Mayumi T. (1991). Amino acids and peptides. XIV. Laminin related peptides and their inhibitory effect on experimental metastasis formation.. Biochem Biophys Res Commun.

[PDF_00740] Kim W. H., Schnaper H. W., Nomizu M., Yamada Y., Kleinman H. K. (1994). Apoptosis in human fibrosarcoma cells is induced by a multimeric synthetic Tyr-Ile-Gly-Ser-Arg (YIGSR)-containing polypeptide from laminin.. Cancer Res.

[PDF_00744] Kramer R. H., Bensch K. G., Wong J. (1986). Invasion of reconstituted basement membrane matrix by metastatic human tumor cells.. Cancer Res.

[PDF_00749] Maeda T., Titani K., Sekiguchi K. (1994). Cell-adhesive activity and receptor-binding specificity of the laminin-derived YIGSR sequence grafted onto Staphylococcal protein A.. J Biochem.

[PDF_00752] Martin G. R., Timpl R. (1987). Laminin and other basement membrane components.. Annu Rev Cell Biol.

[PDF_00746] Matsuzaki A., Janowska-Wieczorek A. (1997). Unstimulated human acute myelogenous leukemia blasts secrete matrix metalloproteinases.. J Cancer Res Clin Oncol.

[PDF_00754] Murata J., Saiki I., Azuma I., Nishi N. (1989). Inhibitory effect of a synthetic polypeptide, poly(Tyr-Ile-Gly-Ser-Arg), on the metastatic formation of malignant tumour cells.. Int J Biol Macromol.

[PDF_00757] Nakahata T., Ogawa M. (1982). Hemopoietic colony-forming cells in umbilical cord blood with extensive capability to generate mono- and multipotential hemopoietic progenitors.. J Clin Invest.

[PDF_00760] Nakajima-Iijima S., Hamada H., Reddy P., Kakunaga T. (1985). Molecular structure of the human cytoplasmic beta-actin gene: interspecies homology of sequences in the introns.. Proc Natl Acad Sci U S A.

[PDF_00763] Nomizu M., Yamamura K., Kleinman H. K., Yamada Y. (1993). Multimeric forms of Tyr-Ile-Gly-Ser-Arg (YIGSR) peptide enhance the inhibition of tumor growth and metastasis.. Cancer Res.

[PDF_00766] Saiki I., Murata J., Iida J., Nishi N., Sugimura K., Azuma I. (1989). The inhibition of murine lung metastasis by synthetic polypeptides [poly(arg-gly-asp) and poly(tyr-ile-gly-ser-arg)] with a core sequence of cell adhesion molecules.. Br J Cancer.

[PDF_00770] Sakamoto N., Iwahana M., Tanaka N. G., Osada Y. (1991). Inhibition of angiogenesis and tumor growth by a synthetic laminin peptide, CDPGYIGSR-NH2.. Cancer Res.

[PDF_00772] Segat D., Pucillo C., Marotta G., Perris R., Colombatti A. (1994). Differential attachment of human neoplastic B cells to purified extracellular matrix molecules.. Blood.

[PDF_00775] Sonnenberg A., Gehlsen K. R., Aumailley M., Timpl R. (1991). Isolation of alpha 6 beta 1 integrins from platelets and adherent cells by affinity chromatography on mouse laminin fragment E8 and human laminin pepsin fragment.. Exp Cell Res.

[PDF_00780] Sterling-Levis K., White L., Trickett A. E., Gramacho C., Pittman S. M., Tobias V. (1993). Heterotransplantation of early B-lineage acute lymphoblastic leukemia using a solubilized attachment matrix (Matrigel).. Cancer Res.

[PDF_00783] Tam J. P. (1988). Synthetic peptide vaccine design: synthesis and properties of a high-density multiple antigenic peptide system.. Proc Natl Acad Sci U S A.

[PDF_00786] Timpl R. (1989). Structure and biological activity of basement membrane proteins.. Eur J Biochem.

[PDF_00788] Tomaselli K. J., Hall D. E., Flier L. A., Gehlsen K. R., Turner D. C., Carbonetto S., Reichardt L. F. (1990). A neuronal cell line (PC12) expresses two beta 1-class integrins-alpha 1 beta 1 and alpha 3 beta 1-that recognize different neurite outgrowth-promoting domains in laminin.. Neuron.

[PDF_00795] Uckun F. M., Manivel C., Arthur D., Chelstrom L. M., Finnegan D., Tuel-Ahlgren L., Irvin J. D., Myers D. E., Gunther R. (1992). In vivo efficacy of B43 (anti-CD19)-pokeweed antiviral protein immunotoxin against human pre-B cell acute lymphoblastic leukemia in mice with severe combined immunodeficiency.. Blood.

[PDF_00803] Yamada Y., Kleinman H. K. (1992). Functional domains of cell adhesion molecules.. Curr Opin Cell Biol.

[PDF_00805] Yan Y., Salomon O., McGuirk J., Dennig D., Fernandez J., Jagiello C., Nguyen H., Collins N., Steinherz P., O'Reilly R. J. (1996). Growth pattern and clinical correlation of subcutaneously inoculated human primary acute leukemias in severe combined immunodeficiency mice.. Blood.

[PDF_00800] van Engeland M., Nieland L. J., Ramaekers F. C., Schutte B., Reutelingsperger C. P. (1998). Annexin V-affinity assay: a review on an apoptosis detection system based on phosphatidylserine exposure.. Cytometry.

